# An Overview of the Effectiveness of Bicycle Helmet Designs in Impact Testing

**DOI:** 10.3389/fbioe.2021.718407

**Published:** 2021-09-27

**Authors:** Javid Abderezaei, Fargol Rezayaraghi, Brigit Kain, Andrea Menichetti, Mehmet Kurt

**Affiliations:** ^1^ Department of Mechanical Engineering, Stevens Institute of Technology, Hoboken, NJ, United States; ^2^ Department of Biomedical Engineering, Stevens Institute of Technology, Hoboken, NJ, United States; ^3^ Biomechanics Section, Mechanical Engineering Department, KU Leuven, Leuven, Belgium; ^4^ BioMedical Engineering and Imaging Institute, Icahn School of Medicine at Mount Sinai, NewYork, NY, United States

**Keywords:** bicycle helmets, concussion, traumatic brain injury, TBI, brain injury risk, mitigation system, impact biomechanics, drop test

## Abstract

Cycling accidents are the leading cause of sports-related head injuries in the US. Conventional bicycle helmets typically consist of polycarbonate shell over Expanded Polystyrene (EPS) foam and are tested with drop tests to evaluate a helmet’s ability to reduce head kinematics. Within the last decade, novel helmet technologies have been proposed to mitigate brain injuries during bicycle accidents, which necessitates the evaluation of their effectiveness in impact testing as compared to conventional helmets. In this paper, we reviewed the literature to collect and analyze the kinematic data of drop test experiments carried out on helmets with different technologies. In order to provide a fair comparison across different types of tests, we clustered the datasets with respect to their normal impact velocities, impact angular momentum, and the type of neck apparatus. When we analyzed the data based on impact velocity and angular momentum clusters, we found that the bicycle helmets that used rotation damping based technology, namely MIPS, had significantly lower peak rotational acceleration (PRA) and Generalized Acceleration Model for Brain Injury Threshold (GAMBIT) as compared to the conventional EPS liner helmets (*p* < 0.01). SPIN helmets had a superior performance in PRA compared to conventional helmets (*p* < 0.05) in the impact angular momentum clustered group, but not in the impact-velocity clustered comparisons. We also analyzed other recently developed helmets that primarily use collapsible structures in their liners, such as WaveCel and Koroyd. In both of the impact velocity and angular momentum groups, helmets based on the WaveCel technology had significantly lower peak linear acceleration (PLA), PRA, and GAMBIT at low impact velocities as compared to the conventional helmets, respectively (*p* < 0.05). The protective gear with the airbag technology, namely Hövding, also performed significantly better compared to the conventional helmets in the analyzed kinematic-based injury metrics (*p* < 0.001), possibly due to its advantage in helmet size and stiffness. We also observed that the differences in the kinematic datasets strongly depend on the type of neck apparatus. Our findings highlight the importance and benefits of developing new technologies and impact testing standards for bicycle helmet designs for better prevention of traumatic brain injury (TBI).

## 1 Introduction

Traumatic brain injury (TBI) is a major cause of death and disability, affecting millions of people every year in the U.S. (Taylor et al., 2017). Sport-related TBIs which annually affects about 300,000 to 3.8 million people in the U.S. makes up a large portion of these TBI cases (Winkler et al., 2016; Taylor et al., 2017).

Even though contact sports such as football have amassed extensive attention from the public and media due to frequent reports of career-ending head injuries (Bland et al., 2020), cycling has contributed the highest number of sports-related head injuries (Coronado et al., 2015). The popularity of cycling has been increasing and the number of bicycle-related injuries (Sanford et al., 2015) and fatalities are growing, correspondingly (Fischer, 2017). According to the American Association of Neurological Surgeons, cycling injuries estimated 85,389 of the 446,788 sports-related head injuries reported in the emergency rooms in 2009 (Healy, 2015; AANS, 2018). Besides being a regular form of exercise or an enjoyable pastime for all age groups, cycling is often used as a daily means of transportation in dangerously crowded cities for many individuals which has made cycling-related head injuries a growing cause of concern nationwide.

In the U.S., a recent study found that only 22% of cyclists who sustained head and neck injuries were wearing helmets during the accident; an overwhelming 78% of cyclists were not wearing proper safety equipment for injury prevention (Scott et al., 2019). As of yet, bicycle helmets are the best strategy to protect the head against severe head and brain injuries (Cripton et al., 2014; Joseph et al., 2017; Olivier and Creighton, 2017; Høye, 2018). According to the Fatality Analysis Reporting System, 62% of cyclists killed in 2019 were not wearing a helmet, 15% were helmeted, and 23% were unknown (FARS, 2019). Therefore, substantial attention has been given to the design of protective equipment for cyclists (Sacks et al., 1991; Karkhaneh et al., 2006). Over the years, bicycle helmet designs have employed similar approaches to combating TBIs and have consistently utilized similar, if not the same, materials. These helmets are usually made up of an external shell and a soft polymeric foam liner (Andena et al., 2016). Expanded Polystyrene (EPS) or Polypropylene (EPP) are common material that have been used in the inner liner (Andena et al., 2016). Traditional EPS liners are primarily designed and manufactured to dampen the impacts and reduce the head impact force (Stigson et al., 2017). conventional bicycle helmets have been shown to mitigate linear acceleration which is a requirement by bicycle helmet safety standards such as U.S. Consumer Product Safety Commission (CPSC), Australian and New Zealand Standard (AS/NZS 2063), EN 1078, Snell Memorial Foundation (*e.g.* B95) and American Society for Testing and Materials (ASTM F1447) (Commision, 1998; Hansen et al., 2013; McIntosh et al., 2013). In these tests, helmets are placed on a headform and dropped onto a steel anvil coated with adhesive-backed 80-grit paper (Commision, 1998; Hansen et al., 2013; McIntosh et al., 2013; Bland, 2019; Bliven et al., 2019; Petersen et al., 2020). The head kinematics during the drop tests are then measured using accelerometers and gyroscopes, which are attached at the center of gravity of the headforms. As outlined in these mandatory safety standards, the linear acceleration of the headform should not exceed a certain threshold (i.e., 300 g outlined in CPSC, 1998, Snell B95 Cheung et al., 2004, and ASTM F1447 Chang, 2003, as well as 250 g outlined in AS/NZS 2512.1, 2009, and Sandberg et al., 2018, EN, 1078, 1997). However, cyclists often fall off their bicycles and impact their heads at angles that are not always direct and usually varies between 30° and 60° (Bourdet et al., 2012; Bourdet et al., 2014). These impacts not only can cause linear acceleration but can also result in rotational acceleration due to the tangential forces to the head (McIntosh et al., 2013; Willinger et al., 2019). Many studies have shown that the rotational acceleration or rotational velocity rather than the linear acceleration are responsible for causing large shear strains in the brain tissue, which could lead to strain concentration (Laksari et al., 2015; Laksari et al., 2018; Abderezaei et al., 2019; Laksari et al., 2020; Mojahed et al., 2020), and potentially result in mild TBI (Holbourn, 1943; Holbourn, 1944; Hardy et al., 2007; Post and Blaine Hoshizaki, 2015; Deck et al., 2019).

Recently, new technologies that are aimed towards mitigating the head’s kinematics through rotation-damping systems have been introduced. These mitigation systems either include spherical slip interfaces (Bliven et al., 2019), and collapsible structures (Hansen et al., 2013; Stigson et al., 2017) in the liner structure, or use a new form of protective gear based on airbag technology (Kurt et al., 2017). Multi-directional Impact Protection System (MIPS) is a relatively new concept that introduces a slip liner inside the helmet; MIPS aims to mitigate rotational impact forces by allowing the head to slide relative to the helmet during the impact (Bottlang et al., 2020). Other technologies, such as WaveCel and Koroyd, utilize a collapsible cellular structure that absorbs the force of impact and minimizes the energy transferred to the cyclist’s head (Hansen et al., 2013; Bliven et al., 2019). Although these advancements are opening the door to the future of cycling safety and TBI prevention, a robust and thorough evaluation of the effectiveness of these novel helmets in mitigating impacts is still incomplete. The aim of this paper is to perform a literature review in PubMed and SCOPUS databases and collect the kinematics of drop test experiments performed on bicycle helmets. We will investigate the kinematic-based injury metrics including peak linear acceleration (PLA), peak rotational acceleration (PRA), and Generalized Acceleration Model for Brain Injury Threshold (GAMBIT) of each new mitigation technology as compared to the conventional helmets. Additionally, the effect of different drop test protocols such as anvil angle, headform position, presence or absence of the neck will be considered in the above analysis.

## 2 Methods

### 2.1 Searching Methodologies and Data Collection

The articles retrieved from the electronic databases PubMed and SCOPUS were selected in a multi-step process. The following key terms were used for PubMed and SCOPUS respectively: **1-** (helmet* AND (cycl* OR bicycle*) AND (drop test* OR impact test* OR impact pendulum test*)), **2-** helmet* AND (cycl* OR bicycle*) AND ((drop test*) OR (impact test*) OR (impact pendulum test*)). After inputting the key terms into each database, the titles and abstracts of each article were manually screened to determine the relevance to the topic of bicycle helmet testing. After excluding irrelevant articles, the full text of each article was reviewed for the following exclusion criteria: 1) Does not perform bicycle helmet drop tests, 2) Does not specify the helmet model, 3) Does not test adult bicycle helmets, 4) Does not test side or front impact performance of the bicycle helmets (since these are the most common impact locations in real-life cycling accidents (Larsen, 1991)), 5) Does not have quantitative information about impact velocity of the drop test, 6) Does not provide quantitative information on kinematic parameters including PLA, and PRA. The date of the last search was May 13, 2021, and the search was restricted to the English language. The inclusion criteria and data extraction of the papers were cross-checked by three independent reviewers.

Having identified all the relevant articles in the two databases, we retrieved the following information for each of the helmet tests from each paper: 1) Type of mitigation technology in the bicycle helmet, 2) PLA, PRA, and PRV, 3) Drop test impact velocity, 4) Anvil angle, 5) Headform model, 6) Presence or absence of the neck surrogate in the headform, 7) Impact location.

### 2.2 Types of Impact Mitigation Technologies in Bicycle Helmets

The helmets collected and analyzed in this paper were mainly organized into two different categories: 1) Conventional helmets, which only use one layer of EPS or Expanded Polypropylene (EPP) as a liner (Table 1; Supplementary Table S1). 2) Helmets with a mitigation system that use one of the following materials or technologies in the liner or the overall design: *MIPS*, *Shear Pad Inside (SPIN)*, *Omni-Directional Suspension (ODS)*, *WaveCel*, *Angular Impact Mitigation (AIM)*, *Koroyd* and *H* $\ddot{o}$ *vding* (Table 1; Supplementary Table S1).

**TABLE 1:** Overview of the literature with relevant kinematic information of the bicycle helmet drop test experiments.

Study	Mitigation type	Headform model	Anvil angle (°)	Impact location	Impact velocity (m/s)	Number of side impact locations(s)^b^	Number of front impact locations(s)^b^
Mills and Gilchrist (2008)	Conventional	Ogle headform w/o the neck^a^	0	Side	4.5	1	1
Hansen et al. (2013)	Conventional, AIM	Magnesium ISO headform on the HIII neck	0, 30	Front	4.8	0	2
Cripton et al. (2014)	Conventional	HIII headform on the ball arm neck	0	Front	5.4, 6.3, 7.7	1	1
Stigson et al. (2017)	Conventional, MIPS, Hövding, Koroyd	HIII headform w/o the neck	45	Side, Front	6	1	1
Kurt et al. (2017)	Conventional, Hövding	NOCSAE headform on the rigid neck	0	Side	6	1	0
Bland et al. (2018a)	Conventional	NOCSAE or HIII headform with and w/o the HIII neck	45	Side, Front	6	1	1
Bland et al. (2018b)	Conventional, MIPS, Koroyd	NOCSAE headform on the HIII neck	30	Side	5.1, 6.6	1	0
Bland et al. (2018c)	Conventional, MIPS, Koroyd	Magnesium ISO headform on the ball arm neck	0	Side	3.4, 6.2	1	0
Bliven et al. (2019)	Conventional, MIPS, WaveCel	HIII headform on the HIII neck	30, 45, 60	Front	4.8, 6.2	0	3
Petersen et al. (2020)	Conventional	NOCSAE headform on the HIII neck	45	Side, Front	6.5	1	1
Bottlang et al. (2020)	Conventional, MIPS, SPIN, ODS	HIII headform on the HIII neck	45	Front	6.19	0	1
Abayazid et al. (2021)	Hövding, SPIN, WaveCel	HIII headform w/o the neck	45	Side, Front	6.3	2	1

a The Ogle headform in Mills and Gilchrist (2008) was connected to a partial neck which was considered in the no-neck group in our analysis.

b Shows the variation of impact locations on the side and front of the helmet.

Conventional bicycle helmets consist of three layers: an ABS plastic outer shell, an EPS or EPP foam liner, and an inner layer of soft foam padding. MIPS seeks to reduce rotational kinematics of the head by permitting sliding between the helmet and head during the impact (Halldin et al., 2003; Bliven et al., 2019; Bottlang et al., 2020). In these helmets, the slip liner that is attached underneath the EPS layer allows for relative motion in all directions and aims to reduce the amount of energy transferred to an individual’s head (Bottlang et al., 2020). SPIN is a technology that replaces comfort padding with silicone padding (Bottlang et al., 2020). These specially developed pads are placed in critical locations in the helmet under the EPS layer and can shear in any direction to produce the same effect as a moving slip liner (Bliven et al., 2019; Abayazid et al., 2021). ODS utilizes two EPS liners that are connected by an array of elastomeric dampers (Bottlang et al., 2020). The array of dampers is designed to support the EPS liners to isolate impact energy from the brain and deflect angular impacts (Bottlang et al., 2020). The collapsible structure mitigation systems we considered in this paper are WaveCel and Koroyd technologies. WaveCel is made from a cellular copolymer material that flexes and glides to absorb energy from impacts and redirect energy away from the head (Bliven et al., 2019; Abayazid et al., 2021). The V-shaped collapsible cellular structure is recessed within the helmet liners and provides rotational suspension (Bliven et al., 2019). Koroyd utilizes thousands of co-polymer extruded tubes that are thermally welded together to create thermo-formed sheets of the helmet liner (Gokhale, 2016). The large compression volumes of the structures create a crumple zone that allows for minimal energy transfer to the head (Gokhale, 2016). AIM is another helmet that uses collapsible structure mitigation system. The AIM system is a non-commercially available cellular structure technology developed by (Hansen et al., 2013). The AIM system replaces EPS by an elastically suspended aluminum honeycomb liner between an inner and outer shell that absorbs linear and angular acceleration (Hansen et al., 2013; Bliven et al., 2019). The honeycomb structure creates a crumple zone that dissipates impact energy through in-plane deformation (Hansen et al., 2013). Additionally, we also considered H $\ddot{o}$ vding, an expandable helmet that uses high-rate micro-electrical-mechanical sensors that can detect a collision and expand to protect the rider’s head before impact (Kurt et al., 2017). Unlike most helmets, H $\ddot{o}$ vding protective gear (all versions including 1, 2, and 3) employs air pressure as a means of protection rather than a typical foam padding (Kurt et al., 2017).

### 2.3 Post Processing of the Extracted Data

In order to provide a fair comparison across different types of tests, we clustered the datasets with respect to their normal impact velocities and impact angular momentums. In the first part, to be able to compare all the extracted headform kinematics whose drop tests were performed at anvil angles ranging from 0° to 60°, an impact velocity clustering step was performed so that the velocity vector would be perpendicular to the anvil:

$$V_{N}=Vcos\theta$$ (1)

where *V*_*N*_ is the impact velocity perpendicular to the anvil plate with angle *θ*.

In the second part, to investigate the effect of headform position and presence or absence of the neck on the rotational acceleration, we clustered the data according to the impact angular momentum *H*_*Impact*_. For more information regarding the calculation of *H*_*Impact*_ please see Supplemental Material.

Next, the K-means algorithm from Python’s machine learning library Scikit-learn (Pedregosa et al., 2011) was used to cluster the data according to *V*_*N*_ and *H*_*Impact*_. For *V*_*N*_, two cluster centers were calculated by using K-means algorithm for low and high *V*_*N*_ and the impact tests with *V*_*N*_ within ±10% of the cluster centers were retained for each group. For *H*_*Impact*_, after removing outliers with *H*_*Impact*_ > 5.2, we calculated one cluster center and the impact tests with *H*_*Impact*_ within ±15% of the cluster center were retained.

The kinematic-based injury metrics including PLA, PRA, and GAMBIT were then compared between the helmets within each group of low and high *V*_*N*_ as well as *H*_*Impact*_. Here, we used GAMBIT since it can be directly calculated from the available kinematics data, and can be used as injury criteria investigating the combined effect of linear and rotational impulses (Newman, 1986; Newman and Shewchenko, 2000; Klug et al., 2015). The GAMBIT value in its general form can be written as:

$$G=max\left({\left[{\left(\frac{a\left(t\right)}{a_{c}}\right)}^{n}+{\left(\frac{\alpha \left(t\right)}{\alpha_{c}}\right)}^{m}\right]}^{1/s}\right)$$ (2)

where *a*(*t*) and *α*(*t*) are translational and rotational accelerations at time *t*, respectively. n, m, and s are empirically derived constant parameters that were fitted to experimental data (Newman and Shewchenko, 2000). *a*_*c*_ and *α*_*c*_ are thresholds derived for a pure translational and rotational acceleration, respectively. Here, we selected n = m = s = 2, a_c_ = 250 g, and *α*_*c*_ = 25,000 rad/s^2^ as was suggested by (Newman and Shewchenko, 2000). It should be noted that when analyzing GAMBIT, *G* = 1 correspond to a 50% probability of Abbreviated Injury Scale (AIS) > 3 which corresponds to serious injury (Newman and Shewchenko, 2000).

### 2.4 Statistical Analysis

In the next step, we investigated the collected drop test results for the following parameters: 1) Presence or absence of the mitigation system, 2) Effect of mitigation type, and 3) Presence or absence of the neck surrogate. To analyze the effect of the presence of the mitigation system, PLA, PRA, and GAMBIT at low and high *V*_*N*_ were compared between the conventional helmets and helmets that used a mitigation system. We then restricted our data to tests that had either included or excluded the neck surrogate in their experiments and performed the same analysis. Finally, the data was clustered according to *H*_*Impact*_ and the effect of mitigation systems and neckform on PRA was analyzed.

Before performing the statistical analysis, we used Shapiro-Wilk’s test to verify the normality of the distribution of the data within each group (Shapiro and Wilk, 1965). We then tested the equal variance of every couple of sample groups considered for the comparisons via Levene’s test (Olkin et al., 1960). We carried out the two-sample t-test if both of the compared groups were normally distributed, otherwise we performed the two-sided Kolmogorov-Smirnov test (Hodges, 1958).

## 3 Results

**FIGURE 1:**
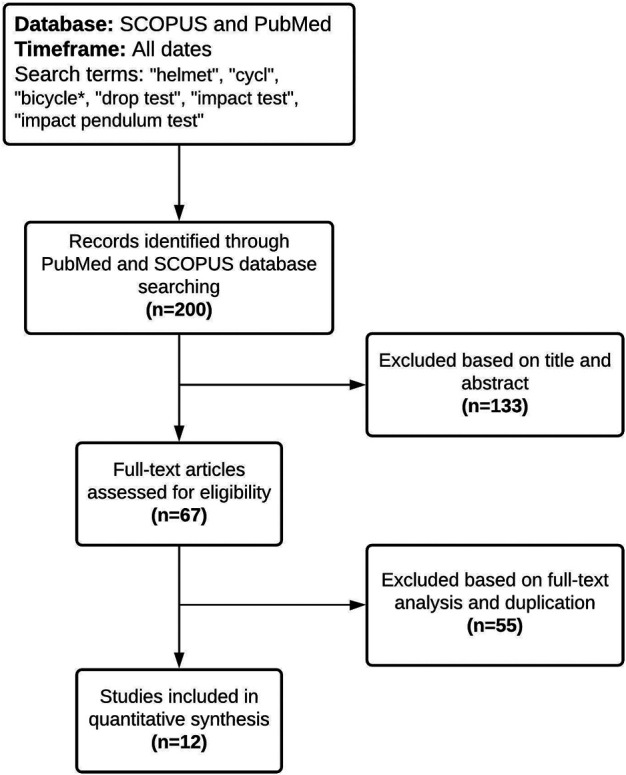
Flowchart outlining the selection of relevant studies.

A flowchart is used to show the procedure of the literature review and the articles that were excluded and included (Figure 1). The PubMed database search resulted in 53 articles pertaining to bicycle helmet testing and the SCOPUS search resulted in 147 articles. Each resulting article was screened and excluded if the title and abstract were not deemed relevant, which resulted in the removal of 133 studies from the data pool. The remaining 67 articles were screened for the necessary inclusion criteria, such as PLA, PRV, PRA (Section 2.1), as well as duplicates. In the end, 12 articles were eligible for inclusion in this review paper (Figure 1; Table 1; Supplementary Table S1).

**FIGURE 2:**
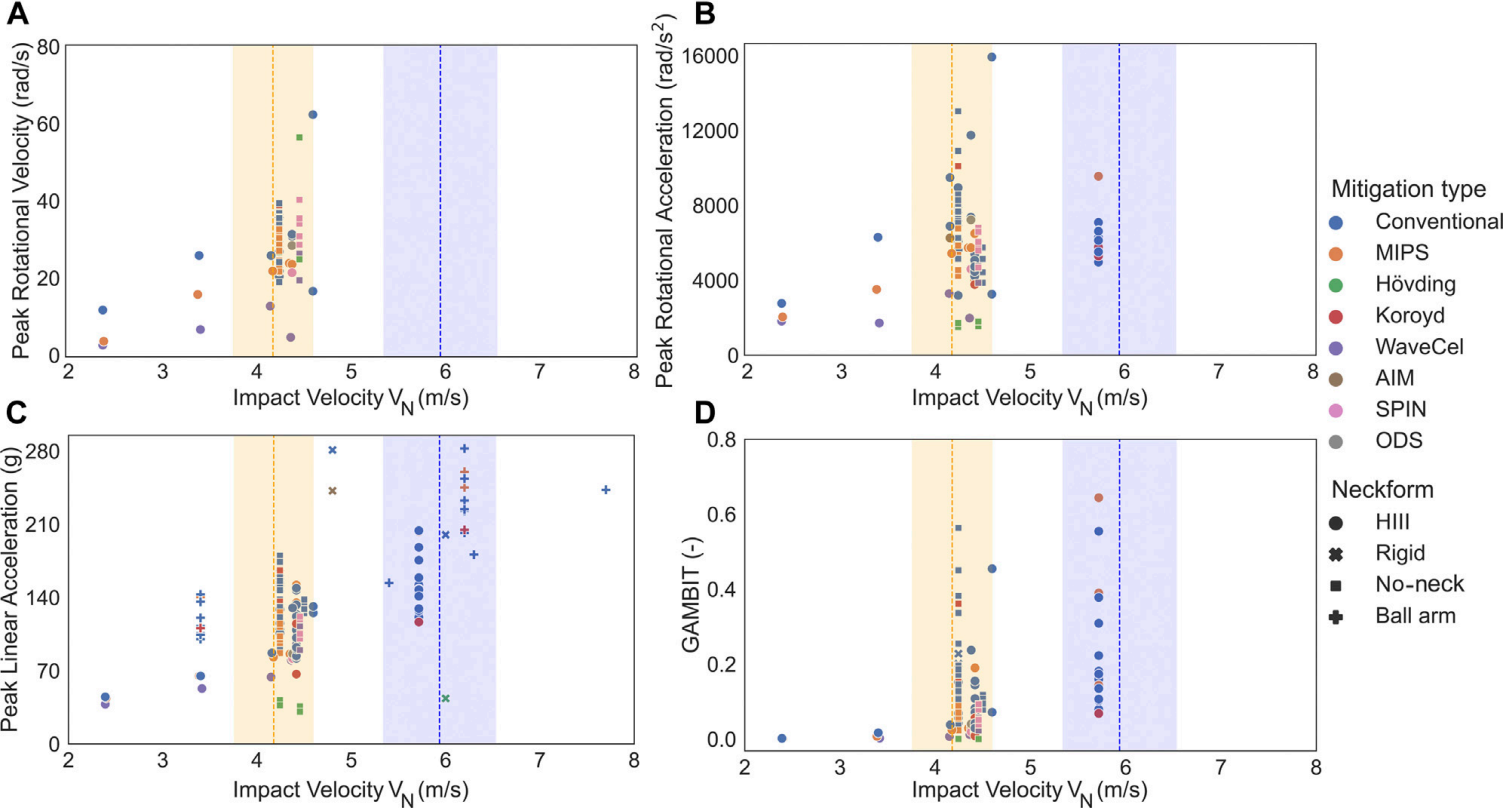
Head kinematics and the GAMBIT value at low and high clustered impact velocity (***V***_***N***_) for all of the extracted bicycle helmets. **(A)** Peak rotational velocity, **(B)** peak rotational acceleration, **(C)** peak linear acceleration, **(D)** and GAMBIT in bicycle helmets with different mitigation technologies which were tested on headforms with or without a neck surrogate. Dashed lines in each figure indicate the cluster centers of low and high *V*_*N*_ and the shaded areas show those impact tests in which the velocities are within 10% of the cluster centers. No data were available in the high *V*_*N*_ range for peak rotational velocity.

A total of 148 bicycle helmet drop tests were collected from the selected papers (It should be mentioned that those data in the studied papers that didn’t pass our criteria, were not included in this review paper). 88 of these helmet drop tests were carried out on the conventional helmets which only used one layer of EPS or EPP as a liner in their design (Figure 2; Table 1; Supplementary Table S1). The remaining 60 of the drop tests were performed on MIPS, SPIN, ODS, WaveCel, AIM, and Koroyd helmets and H $\ddot{o}$ vding protective gear (Figure 2; Table 1; Supplementary Table S1). The impact velocities of the tests varied between 3.4 m/s and 7.7 m/s. After applying the k-mean clustering algorithm (Pedregosa et al., 2011), we found *V*_*N*_ = 4.2 m/s and *V*_*N*_ = 5.9 m/s to be the cluster centers of low and high impact velocities, respectively (Figure 2; Table 1; Supplementary Table S1). Impact tests outside the 10*%* of the cluster centers were then removed, resulting in 75 conventional and 51 mitigation type helmet drop tests. Among the studied literature for this paper, four different types of neck-headform attachments were observed: **1-** No neckform was attached to the head (N in Figure 2), **2-** The headform was attached to a ball-arm neck (Ball arm in Figure 2), **3-** The headform was attached to a rigid neck (Rigid in Figure 2), and **4-** The headform was attached to a Hybrid III 50th-percentile male neck (Y in Figure 2).

**FIGURE 3:**
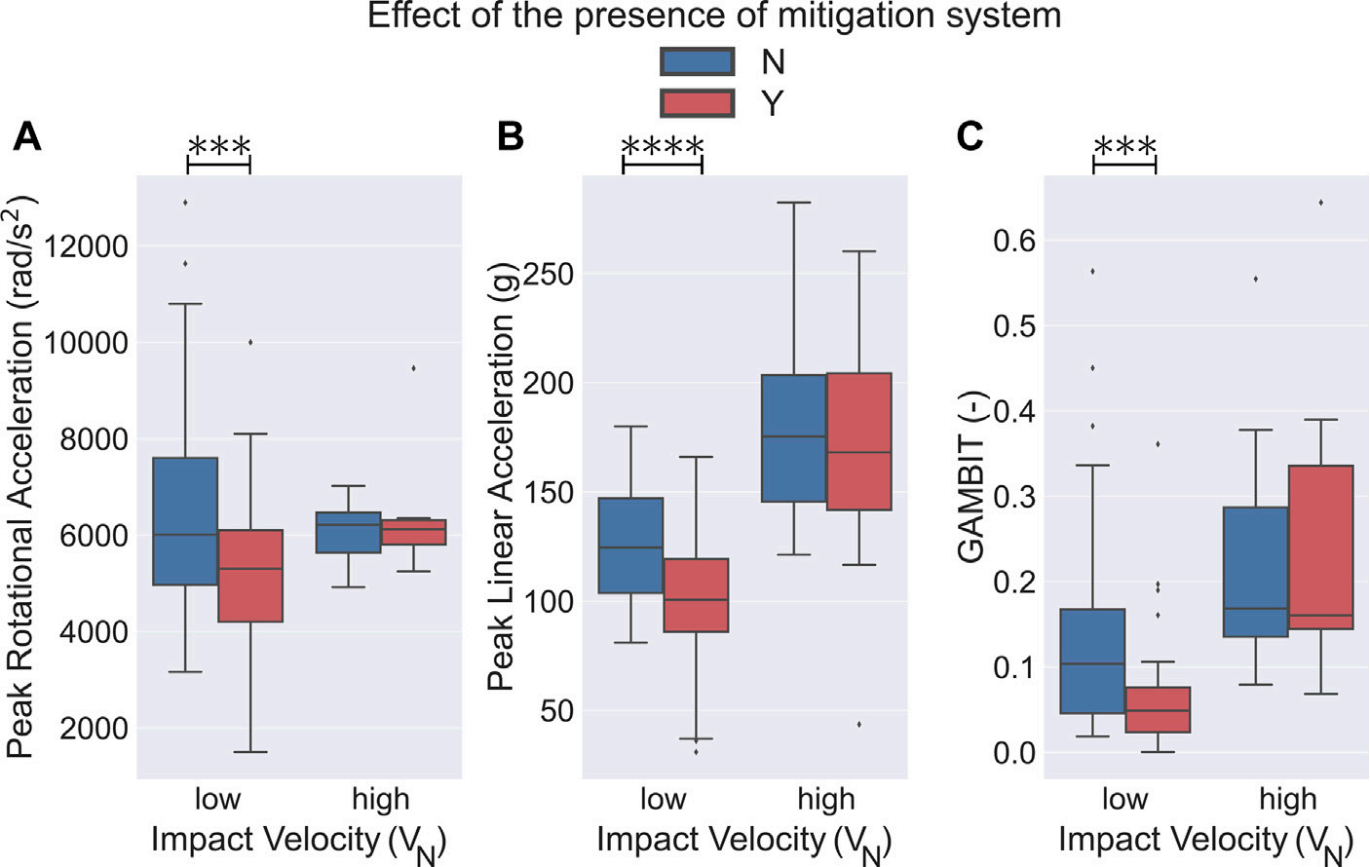
Effect of the presence or absence of the mitigation system on bicycle helmet performance in impact tests. Helmets using a mitigation technology had a significantly lower **(A)** PRA, **(B)** PLA, and **(C)** low *V*_*N*_ as compared to the conventional helmets (*p* <0.001). No statistical significance was observed in high *V*_*N*_ (5.9 ± 0.6 m/s) drop tests between the two different helmet types. ⧫ shows the outlier data.

Having collected all the existing bicycle helmet drop test results from the literature survey, we first analyzed the effect of the presence or absence of the impact mitigation systems on the resultant kinematics and the associated injury metrics during drop tests (Figure 3). We observed that at low *V*_*N*_ (4.2 ± 0.4 m/s) drop tests, the bicycle helmets with a mitigation system, on average, had significantly lower PLA, PRA, and GAMBIT values compared to conventional helmets (approximately 20.2, 21.8, and 52.6*%* lower respectively, Figures 3A–C, *p* < 0.01). Here, the low *V*_*N*_ (4.2 ± 0.4 m/s) drop test experiments of the bicycle helmets with a mitigation system resulted in average PLA, PRA, and GAMBIT of 100.1 ± 30.4 m/s, 5,043.6 ± 1740.8 *rad*/*s* ^2^, and 0.062 ± 0.066, respectively. The conventional bicycle helmets, on the other hand, experienced an average PLA, PRA and GAMBIT of 125.5 ± 26.9 m/s, 6,448.8 ± 1985.6 rad/*s* ^2^, and 0.131 ± 0.111, respectively. In the drop tests at high *V*_*N*_ (5.9 ± 0.6 m/s), we did not observe any statistically significant differences between the kinematics of the bicycle helmets with and without the mitigation systems (Figures 3A–C). For these experiments, we observed average PLA, PRA, and GAMBIT values of 169.5 ± 61.0 m/s, 6,504.7 ± 1,370.0 rad/s^2^, and 0.261 ± 0.198, for the helmets with a mitigation system, respectively. The experiments on the conventional helmets resulted in average PLA, PRA, and GAMBIT values of 179.6 ± 41.6 m/s, 6,075.7 ± 548.9 rad/s^2^, and 0.215 ± 0.126, respectively.

**FIGURE 4:**
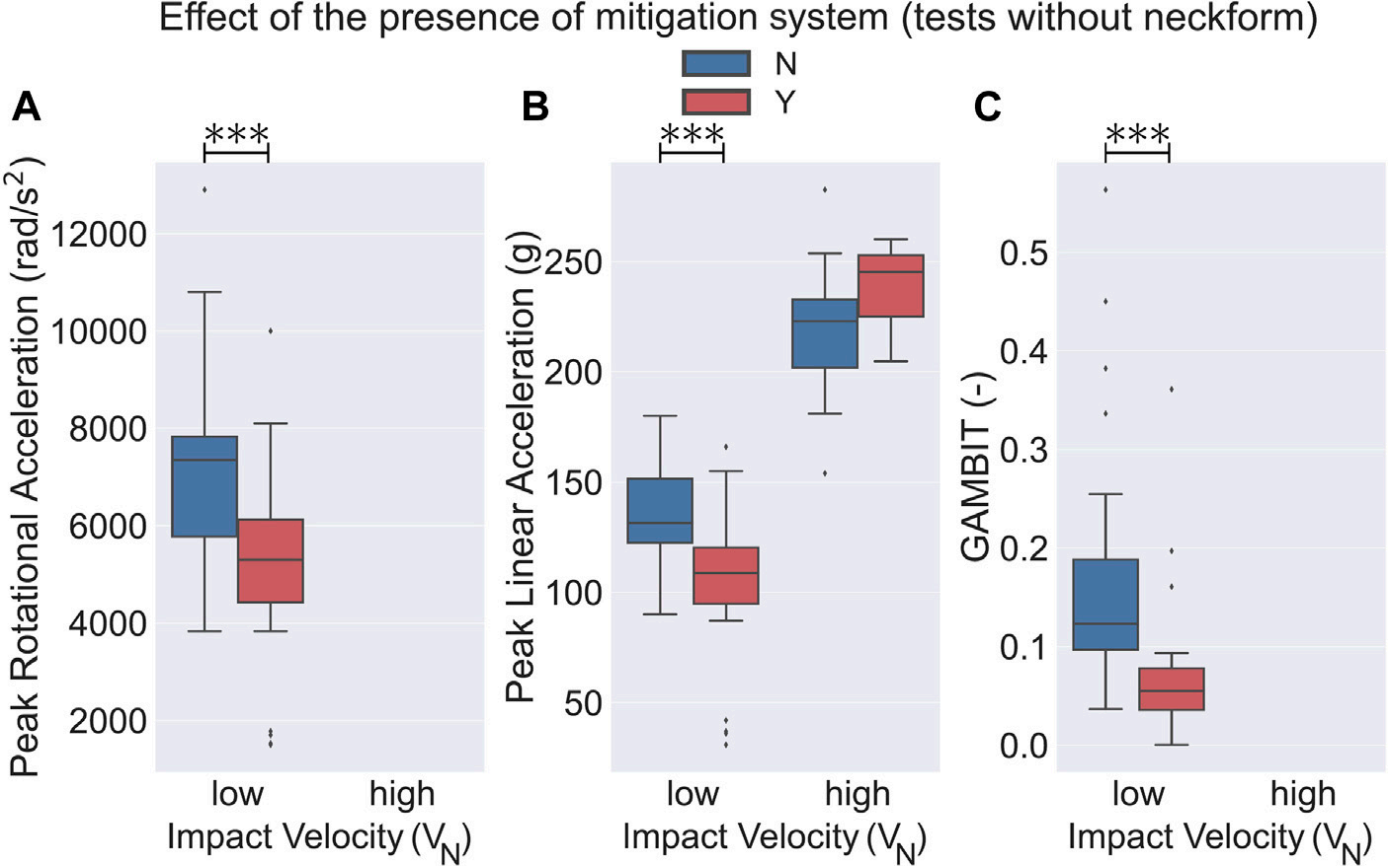
Effect of the presence of the mitigation system on bicycle helmets that were tested on headforms without a neck surrogate. Helmets with a mitigation technology had a significantly lower **(A)** PRA (*p* < 0.001), **(B)** PLA (*p* < 0.001), and **(C)** GAMBIT (*p* < 0.001) in drop tests at low *V*_*N*_ (4.2 ± 0.4 m/s). No statistical significance was observed in PLA at high *V*_*N*_ (5.9 ± 0.6 m/s) drop tests. In high *V*_*N*_ drop tests, no data were available for PRA and GAMBIT. ⧫ depicts the outlier data.

**FIGURE 5:**
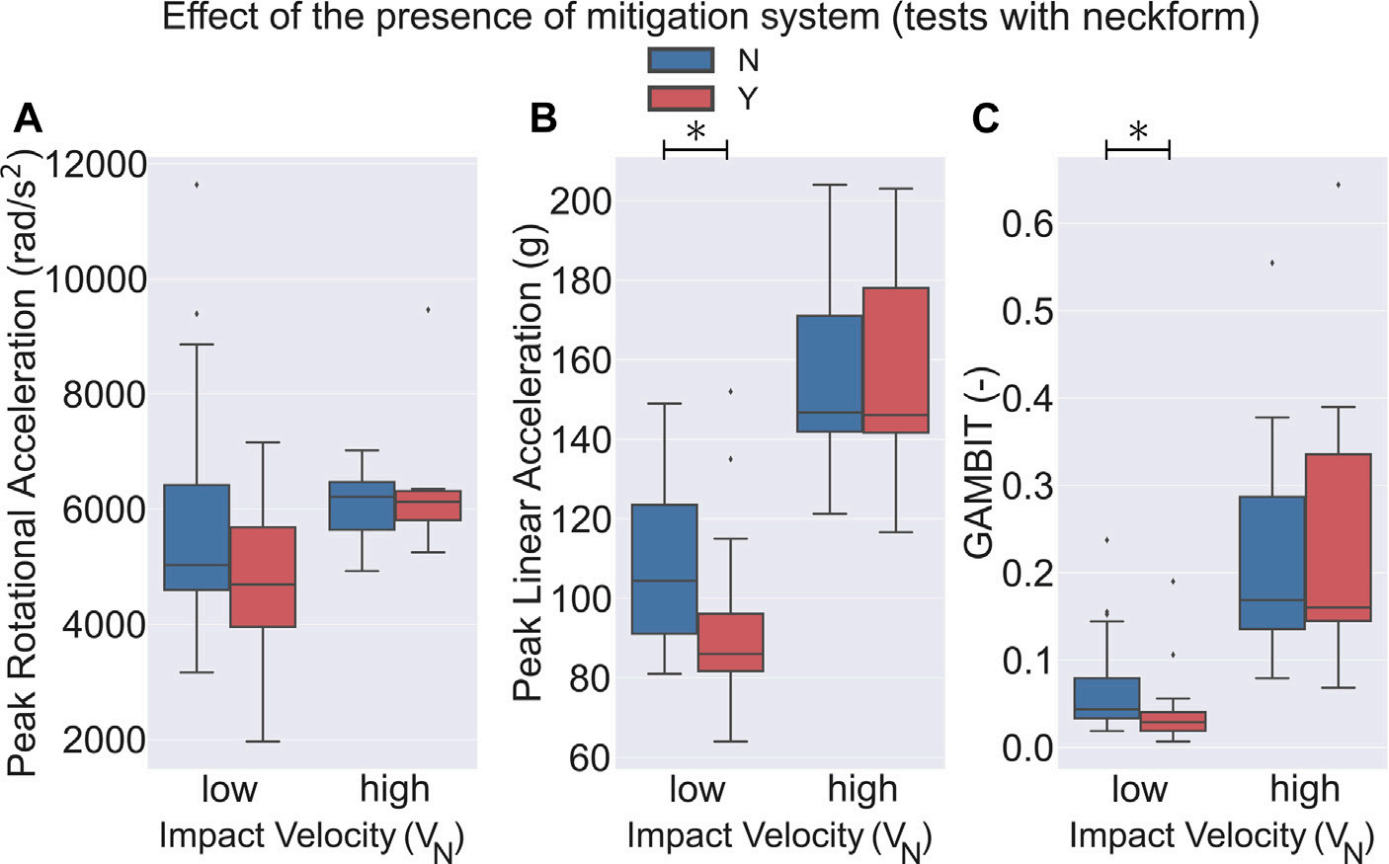
Effect of the presence of the mitigation system on bicycle helmets that were tested on headforms with a neck surrogate. **(A)** No statistical significance was observed in PRA between the two groups at both low and high *V*_*N*_ (5.9 ± 0.6 m/s) drop tests of neck included groups. Helmets with a mitigation system had a significantly lower **(B)** PLA and **(C)** GAMBIT at low *V*_*N*_ as compared to the conventional helmets (*p* < 0.05). No statistical significance was observed for PLA and GAMBIT at high *V*_*N*_ (5.9 ± 0.6 m/s). ⧫ depicts the outlier data.

One crucial difference in the different drop tests we considered for this paper was the presence or absence of the neck surrogate. We found that 65 experiments were performed on headforms with an attached neck surrogate and the remaining 52 were tested on headforms without a neck component. 8 helmets were tested with a rigid neck attached to the headform and 23 were tested while being attached to a ball arm. In our analysis, we considered the headforms attached to a ball arm in the no-neck group since in both of these groups the headform could rotate without resistance at the time of the impact. Our first finding was that in almost all of the categories, tests without a neck component experienced a higher PLA, PRA, and GAMBIT on average as compared to the group with an attached neck component (Figures 4, 5). Here, in the low *V*_*N*_ (4.2 ± 0.4 m/s) drop tests, PLA, PRA, and GAMBIT, on average, were approximately 10.3, 7.3, and 59.3% higher in the no-neck group, respectively. At high *V*_*N*_ (5.9 ± 0.6 m/s) drop tests, PLA was on average 51.0% higher in the no-neck group. It should be noted that no PRA values were available at high *V*_*N*_ (5.9 ± 0.6 m/s) drop tests for the no-neck group. Next, we analyzed the effect of the presence of an impact mitigation system in each of the neck and no-neck groups. We observed that for the low *V*_*N*_ (4.2 ± 0.4 m/s) tests, in the no-neck group the bicycle helmets with a mitigation system had a significantly lower PLA (24.7*%*), PRA (27.5*%*), and GAMBIT (59.7*%*) as compared to the conventional bicycle helmets (Figure 4, *p* < 0.001). Whereas, in the neck-included group, only PLA (13*%*) and GAMBIT (36.2*%*) were significantly lower in the helmets with a mitigation system (Figure 5, *p* < 0.05). Additionally, we did not observe any statistically significant differences of PLA between the helmet models at high *V*_*N*_ (5.9 ± 0.6 m/s) drop tests. No data points were available for PRA and GAMBIT in the no-neck group at high *V*_*N*_ (Figures 4A–C)).

**FIGURE 6:**
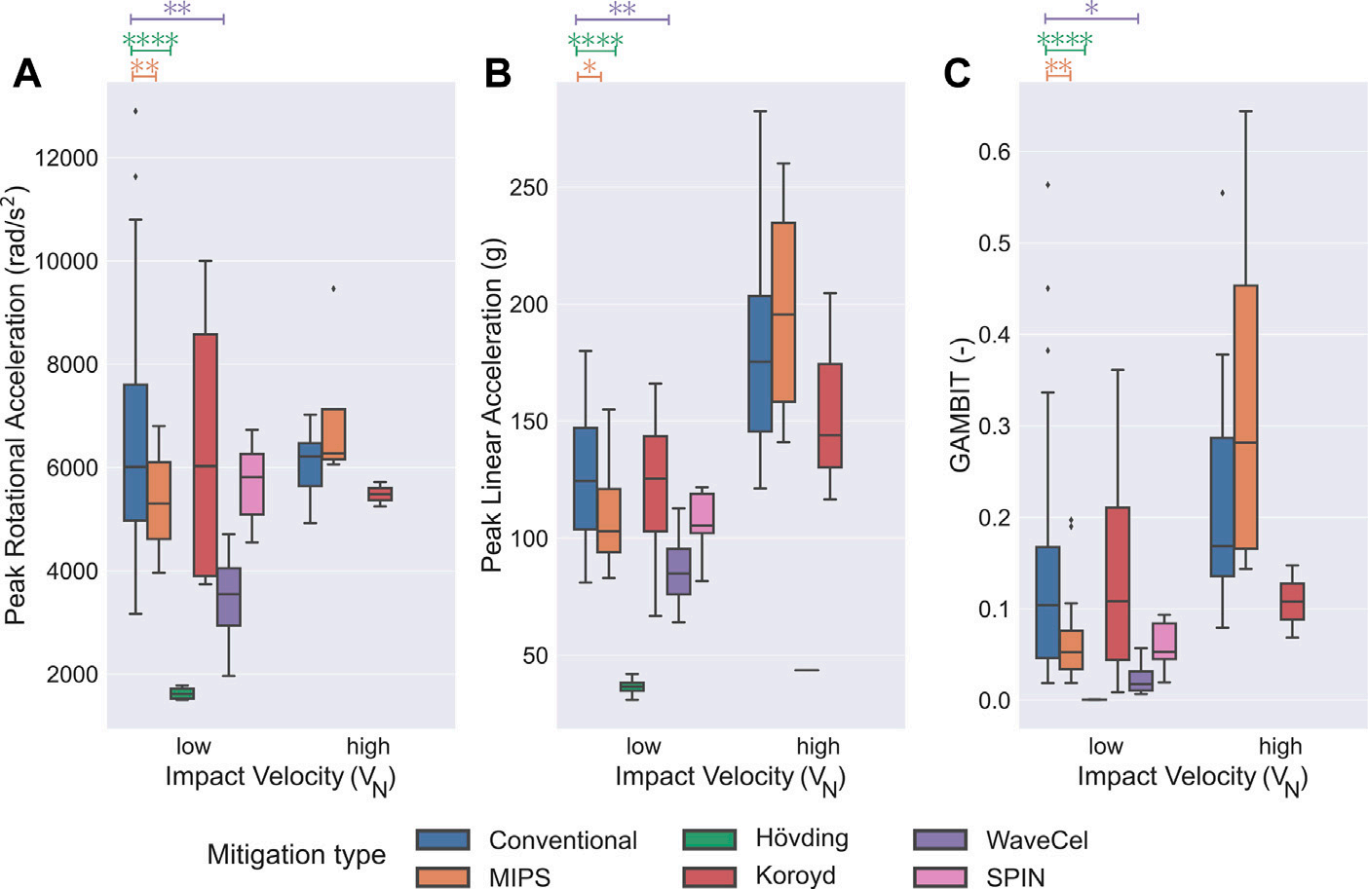
Effect of different mitigation systems in drop tests at low and high *V*_*N*_s. **(A)** Compared to the conventional bicycle helmets, PRA was significantly less in WaveCel (*p* < 0.0001), SPIN (*p* < 0.05), H $\ddot{o}$ vding (*p* < 0.001) and MIPS (*p* < 0.05) at low *V*_*N*_ (4.2 ± 0.4 m/s) drop tests. **(B)** Compared to the conventional bicycle helmets, PLA was significantly less in H $\ddot{o}$ vding (*p* < 0.0001) and WaveCel (*p* <0.05) in drop tests at low *V*_*N*_ (4.2 ± 0.4 m/s). **(C)** GAMBIT was significantly less in H $\ddot{o}$ vding (*p* < 0.001), WaveCel (*p* < 0.05) and SPIN (*p* < 0.05) compared to the conventional ones in low *V*_*N*_ (4.2 ± 0.4 m/s) drop tests. No statistically significant differences were observed at high *V*_*N*_ (5.9 ± 0.6 m/s) drop tests between the conventional helmets and other technologies. In this figure, only technologies with at least 4 data points were included. ⧫ depicts the outlier data.

In the next step, we investigated the efficacy of the different mitigation technologies by comparing PRA, PLA, and GAMBIT of each specific mitigation technology with conventional bicycle helmets (Figure 6). Here, we only considered helmet types with at least 4 data points for the comparison. We found that among the helmets that used rotation-damping based technologies, only MIPS had approximately 16.8 and 49.3*%* lower PRA and GAMBIT at low *V*_*N*_ (4.2 ± 0.4 m/s) as compared to the conventional helmets, respectively (Figures 6A,C, *p* < 0.05). While SPIN helmets had on average lower PLA, PRA, and GAMBIT of about 14.5*, 11.9*, and 53.8*%*, respectively, we did not find any statistically significant differences in these helmets as compared to the conventional ones. Next, we analyzed the effectiveness of helmets that used collapsible structures in their liner. In this category, helmets based on the WaveCel technology had a significantly lower PLA, PRA, and GAMBIT of approximately 31.0, 46.6, and 81.1*%* at low *V*_*N*_ (4.2 ± 0.4 m/s) as compared to the conventional helmets, respectively (Figures 6A–C), *p* < 0.05). Whereas, Koroyd which is another helmet based on collapsible structures did not show any statistical differences compared to the conventional ones (Figure 6A–C), *p* < 0.05). Compared to the investigated helmets in the literature, the H $\ddot{o}$ vding protective gear had the best performance in the analyzed kinematic based injury metrics with PLA, PRA, and GAMBIT of about 70.9, 74.8, and 99.5*%* lower than the conventional helmets (*p* < 0.0001). At high *V*_*N*_ (5.9 ± 0.6 m/s), we observed no statistical significance when we compared PRA, PLA, and GAMBIT between the conventional and each of the other helmet types (Figurse 6A–C)). It should also be noted that, for high *V*_*N*_ (5.9 ± 0.6 m/s) we did not have data points for PRA, and GAMBIT values of SPIN, WaveCel, and H $\ddot{o}$ vding protective gears. Moreover, the Koroyd helmets only had two data points at high *V*_*N*_ (5.9 ± 0.6 m/s) experiments for PRA, and GAMBIT, therefore, were not compared with the conventional helmets in this category.

**FIGURE 7:**
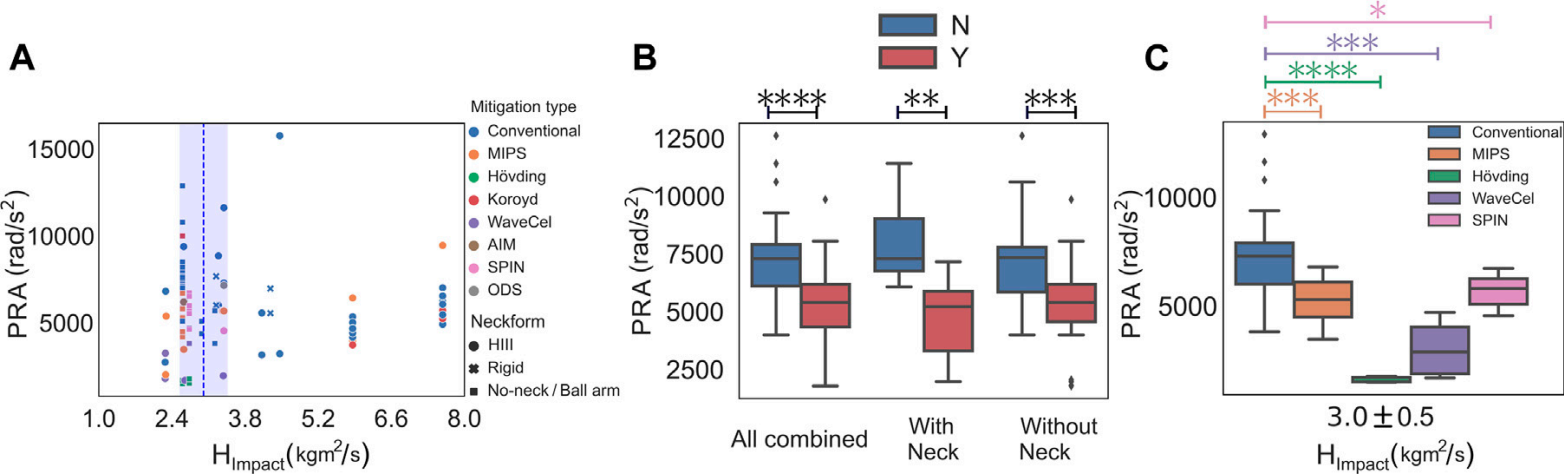
Effect of the presence of mitigation systems on the bicycle helmets after normalization of the data with respect to the impact angular momentum *H*_*Impact*_. **(A)** Peak rotational acceleration of the helmets with different mitigation technologies clustered with respect to *H*_*Impact*_. **(B)** Presence of the mitigation system on bicycle helmets that were tested on headforms with and without a neck surrogate showed significantly less PRA as compared to the conventional helmets (*p* < 0.001). **(C)** Compared to the conventional bicycle helmets, PRA was significantly less in WaveCel (*p* < 0.001), SPIN (*p* < 0.05), H $\ddot{o}$ vding (*p* < 0.0001) and MIPS (*p* < 0.001).

To take into account the effect of headform orientation at the time of impact, as well as the presence or absence of the neckform, we clustered the data according to the impact angular momentum (*H*_*Impact*_) and checked the rotational acceleration of the helmets (Figure 7). After removing the outlier data (*H*_*Impact*_ > 5.2 kgm^2^/s) we found *H*_*Impact*_ = 3.0 ± 0.5 kgm^2^/s to be the cluster center. This narrowed the data from the literature to 79 tested helmets within the range of *H*_*Impact*_ = 3.0 ± 0.5 kgm^2^/s (Figure 7A). In the next step, we analyzed the effect of the mitigation system on PRA of the headforms. We observed that within *H*_*Impact*_ = 3.0 ± 0.5 kgm^2^/s, the PRA of the helmets that used a mitigation system was approximately 31.0*%* lower compared the conventional helmets (*p* < 0.0001; Figure 7B). Next, we separately analyzed the PRA of the groups with and without the neckform. We observed in both of these two groups that the PRA of the helmets with a mitigation system was significantly lower compared to the conventional helmets (*p* < 0.01; Figure 7B). Finally, within the range of *H*_*Impact*_ = 3.0 ± 0.5 kgm^2^/s, we analyzed the performance of each of these individual mitigation technologies against the conventional helmets. For this analysis, we only considered mitigation technologies with at least 4 data points. We observed that within this *H*_*Impact*_ range, H $\ddot{o}$ vding protective gear had the best performance, with a lower PRA of approximately 78*%* in comparison to the conventional helmets (*p* < 0.0001; Figure 7C). WaveCel with a lower PRA of about 58*%* as compared to the conventional helmets was the next best performing technology in PRA that followed H $\ddot{o}$ vding (*p* < 0.001; Figure 7C). Helmets with a dedicated rotation-damping technologies including MIPS and SPIN also had a significantly lower PRA of approximately 27*%* (*p* < 0.001) and 22*%* (*p* < 0.05) as compared to the conventional helmets, respectively (Figure 7C).

## 4 Discussion

The recent developments in bicycle helmet design technologies have been promising for the future of cycling safety and TBI prevention. In this paper, we performed a literature review on the recent advancements and improvements of these new bicycle helmets and analyzed their performance in reducing the head kinematics compared to the conventional designs. To do so, we extracted kinematic datasets of more than 140 helmet drop tests from the retrieved articles and investigated several kinematics-based injury metrics including PLA, PRA, and GAMBIT.

Overall, we observed that the new protective gear technologies including MIPS, WaveCel, and H $\ddot{o}$ vding significantly decreased PLA, PRA, and the GAMBIT value of the headform at low *V*_*N*_ (4.2 ± 0.4 m/s) drop tests. While the bicycle helmets based on MIPS had a significantly lower PRA, PLA and GAMBIT as compared to the conventional helmets, no statistical differences was observed for the SPIN helmets. The significantly lower PRA values of the MIPS helmets could be due to the dedicated rotation-damping systems in these helmets. In these helmets, the rotational damping mechanism works by adding slip liners underneath the main EPS liner, which allows sliding between the head and the helmet during the impact (Halldin et al., 2003; Bland et al., 2018b; Bottlang et al., 2020). Additionally, the improved PLA response of the MIPS helmets might be due to the improved design and manufacturing quality of these helmets such as the changes in the thickness of EPS padding and the helmets weight. These encouraging findings, highlight the benefit of including rotation damping technologies in helmets in order to reduce the TBI risk during cycling accidents. It should be noted that the lack of statistical differences for the PRA values of the SPIN helmets could be due to grouping the data only according to their impact velocity, which might cause some errors and will be discussed further below.

Next, we investigated the kinematics of other recently developed bicycle helmets based on collapsible structure mitigation systems including WaveCel and Koroyd (Stigson et al., 2017; Bland et al., 2018b; Bland et al., 2018c), our analyses were inconclusive. While the WaveCel helmets performed significantly better than the conventional helmets in linear and rotational kinematics, and the consequential brain injury risk at low *V*_*N*_ (4.2 ± 0.4 m/s) drop tests, the Koroyd based helmets did not show any statistical differences. One of the reasons for the observed kinematics of the Koroyd helmets is potentially due to the low number of available data points. Only 6 drop test results from 3 different Koroyd helmets were available in the literature. When we investigated the performance of each of these helmets, we observed that one of the Koroyd helmets had a significantly better performance than the conventional ones, whereas, the other two had either the same level or much worse performance in the metrics considered. This shows that in addition to incorporating the new technologies in a helmet, it could be important to optimize the conventional helmet design parameters such as weight and liner thickness. The WaveCel helmets, on the other hand, performed consistently better than all others except for H $\ddot{o}$ vding. The significant reduction of PLA as compared to the conventional helmets suggests that the buckling of WaveCel’s organized cellular structure might attenuate radial forces better than the commonly used EPS material (Bland et al., 2018b; Bliven et al., 2019). The significant mitigation of PRA by these helmets could be due to the folding properties of its cellular structure (Bliven et al., 2019). First, each cell can deform tangentially which allows absorption of the shear force between the head and the helmet (Bliven et al., 2019). Second, these cells can also have an elastic in-plane deformation allowing a rotational suspension that decouple the head from the helmet (Bliven et al., 2019). Overall, in addition to the impact performance of WaveCel helmets in the mitigation of kinematic based injury metrics, advantages such as its light weight, high heat transfer rate, and airflow permeability, make such honeycomb based helmets potentially a good candidate to replace the conventional EPS/EPP helmets (Caserta et al., 2011; Caccese et al., 2013; Hansen et al., 2013; Bliven et al., 2019).

The H $\ddot{o}$ vding protective gear had a lower PLA, PRA, and GAMBIT as compared to the other helmets. The reasons for this performance stem from the helmet’s large size and low stiffness (Kurt et al., 2017; Abayazid et al., 2021). Such properties result in an increased duration of the impact and significantly lower peak acceleration values (Kurt et al., 2017; Abayazid et al., 2021). Despite the substantial mitigation of PLA and PRA, it should be noted that the prolonged duration of the impact could potentially result in a high PRV, which could carry increased injury risks (Ommaya and Hirsch, 1971; Margulies and Thibault, 1992; Rowson et al., 2012; Hernandez et al., 2015a; Abayazid et al., 2021). Additionally, the H $\ddot{o}$ vding’s large size and its increased duration of the impact, could mean increased coupling of the neck and shoulder during real-life impacts (Abayazid et al., 2021). Therefore, further tests regarding the potential neck injuries (as with any newly developed technology that might introduce such injuries), and other relevant TBI metrics are a necessary step before the widespread use of this type of helmet (Kurt et al., 2017). For instance, due to the lack of standard testing procedures, this type of helmet cannot be sold in the U.S. market (Kurt et al., 2017). However, regardless of these factors, the existing kinematic data highlight the potential of these airbag type bicycle helmets in mitigating the risks of TBI.

Having analyzed the effect of the mitigation systems after grouping the data with respect to the normal impact velocity *V*_*N*_, we also analyzed the effect of headform positioning at the time of the impact. In the experiments gathered from the literature, the helmets have been dropped at various angles of 0–90° on anvils with varying angles of 0–60°. The differences in the impact location of the headform could result in increased or decreased PRA. To analyze this effect, the data was also clustered with respect to the impact angular momentum *H*_*Impact*_. Similar to our previous observations, we found that the helmets with the mitigation technologies still had a significantly lower PRA as compared to the conventional ones (Figure 7). Interestingly, we observed that while the SPIN helmets did not have statistically different PRA compared to the conventional helmets in the normal low *V*_*N*_ group, in the new *H*_*Impact*_ group, they had a significantly lower PRA. This suggests that in the initial grouping according to the low *V*_*N*_, some of the drop tests might have been performed at an angle that caused high angular momentum and high PRA values.

Our analyses of the kinematics data from the literature demonstrate the necessity of taking new steps toward the standardization of bicycle helmet testing procedures. We observed that the presence or absence of the neckform in the drop test experiment affected the recorded kinematics. Initially we grouped the data according to their impact velocity *V*_*N*_ (Figures 4, 5). We observed that at low *V*_*N*_ for the no-neck group, the bicycle helmets with the mitigation system showed a significant reduction of the PLA, PRA,and GAMBIT. Whereas, in the neck included group, there were only statistical differences in the PLA, and GAMBIT values (Figures 4, 5). Additionally, the PRA, PLA, and GAMBIT were substantially larger in the no-neck group. The observed lack of statistical significance of PRA in the neck included group could be due to the absorption of part of the rotational kinematics by the stiff neck (Hernandez et al., 2015b). It has been shown in laboratory testing that the Hybrid III neck surrogate (the most commonly used neck model in the analyzed studies, Table 1; Supplementary Tables S1,S2) produces impact dynamics with a higher damping factor and lower natural frequency as compared to real-world impacts (Gwin et al., 2010; Hernandez et al., 2015b). Due to this slowing of the dynamics (Gwin et al., 2010), the mitigation systems might become less engaged in decreasing the head kinematics. Others have also reported similar findings, where the presence or absence of the neck surrogate could result in markedly different kinematics (Hering and Derler, 2000; Bartsch et al., 2012; Camarillo et al., 2013; Bland et al., 2018a), with significantly larger PLA, PRV, and PRA in the no-neck tests of the same helmets (Bland et al., 2018a). Another interesting observation we had was with regards to the presence of the neckform in the impact velocity (*V*_*N*_) and impact angular momentum *H*_*Impact*_ cluster analyses. While the PRA comparisons in the *V*_*N*_ cluster analysis strongly depended on the presence of the neckform (Figures 4, 5), this dependence was not observed in the *H*_*Impact*_ cluster analysis (Figure 7B). These findings, further highlight the importance of standardized testing and analysis of helmet drop tests.

Our results are subject to several limitations. The experimental drop tests in the literature are performed at various heights which result in different impact velocities across the studies. To address this issue, we applied k-mean clustering algorithm to the extracted data and selected two cluster centers and 10% of their surrounding as the impact velocities of interest. This allowed removing outlier data which might have affected the findings because of their high or low impact velocities. To correct for the effect of impact location on the headform which might affect PRA, we also created another group according to the impact angular momentum with one cluster center and 15% standard deviation. Another limitation of our study is the lack of enough data points for some of the compared categories. This was more evident in the lack of PRA values of the drop tests at high *V*_*N*_ (5.9 ± 0.6 m/s) performed without a neck surrogate, as well as, lack of sufficient kinematic data for some of the newly developed helmet technologies. In our results, we observed no statistical significance in the effect of mitigation system for high *V*_*N*_ (5.9 ± 0.6 m/s) tests, which could mainly be due to the lack of enough data points in that testing category. Moreover, in the literature we observed that the drop tests were carried out at various configurations such that the headform and anvil had relative angles in the range of 0–90°. These differences in the experimental procedures could lead to increased or decreased PLA and PRA between similar helmets that were tested in different configurations. To address this limitation we clustered the data according to normal impact velocity (*V*_*N*_) *and impact angular momentum* (*H*_*Impact*_), which allowed comparison of these helmets with each other. Additionally, it should be noted that, here, we analyzed different mitigation technologies across various helmets. A more accurate analysis would be to do this investigation on the same helmets under the same impact conditions, with or without the specific technologies. As such, other parameters such as the liner thickness, helmet mass, presence or absence of the neck surrogate (Fahlstedt et al., 2016; Bland, 2019; Fahlstedt et al., 2021), as well as the headform model (Kendall et al., 2012; Cobb et al., 2016; Bland, 2019) might also confound the interpretation of these results significantly.

## 5 Conclusion

With the introduction of various new bicycle helmet technologies in the last decade, there is a dire need to compare their efficacy in reducing head kinematics with respect to the commonly used conventional bicycle helmets. In this work, we reviewed the literature to collect and analyze various bicycle helmet technologies, by investigating their resultant kinematic-based head injury data from drop test experiments. We observed that the helmets that used new technologies such as rotation damping systems, collapsible cellular structures, and expandable models, performed significantly better than the conventional helmets for kinematics-based metrics at low impact velocities and low impact angular momentum. Additionally, we observed that presence or absence of the neck surrogate in the experimental procedure could result in different kinematics. These findings highlight the importance of rethinking conventional helmet designs, consideration of novel technologies for better prevention of cycling-related TBIs, and the need for more thorough evaluation and impact testing of bicycle helmets.
