# Supplementary material for: An Overview of the Effectiveness of Bicycle Helmet Designs in Impact Testing
Source: Front Bioeng Biotechnol. 2021 Sep 27;9:718407. doi: 10.3389/fbioe.2021.718407 (PMC8503260; doi:10.3389/fbioe.2021.718407)
Supplement: Supplementary file 1 [file DataSheet1.pdf]

Supplemental Material
An overview of the effectiveness of bicycle helmet designs in impact testing

Javid Abderezaei,[1,*] Fargol Rezayaraghi,[1,*] Brigit Kain,[2,*] Andrea Menichetti,[3] and Mehmet Kurt[1,4]

[1]*Department of Mechanical Engineering, Stevens Institute of Technology, Hoboken, NJ, USA*
[2]*Department of Biomedical Engineering, Stevens Institute of Technology, Hoboken, NJ, USA*
[3]*Biomechanics Section, Mechanical Engineering Department, KU Leuven, Leuven, Belgium*
[4]*BioMedical Engineering and Imaging Institute, Icahn School of Medicine at Mount Sinai, New York, NY, USA*
[*] *These authors contributed equally to this work*

Table S1: Overview of the literature with relevant kinematic information of the bicycle helmet drop test experiments.

Study	Helmet Model	Mitigation Type	Headform Model	Anvil Angle (°)	Impact Location	Impact Velocity (m/s)
Mills et al [1]	Bell Avanti	Conventional	Ogle headform w/o the neck	0	Side, Front	4.5
Mills et al [1]	Bell Arc	Conventional	Ogle headform w/o the neck	0	Side, Front	4.5
Hansen et al [2]	Nutcase	Conventional	Magnesium ISO headform on the HIII neck	0, 30	Front	4.8
Hansen et al [2]	Nutcase with AIM	AIM	Magnesium ISO headform on the HIII neck	0, 30	Front	4.8
Cripton et al [3]	CCM V15 Backtrail	Conventional	HIII headform on the ball arm neck	0	Front	5.4, 6.3, 7.7
Stigson et al [4]	Abus S-Force Peak	Conventional	HIII headform w/o the neck	45	Side, Front	6
Stigson et al [4]	Bell Stoker MIPS	MIPS	HIII headform w/o the neck	45	Side, Front	6
Stigson et al [4]	Biltema helmet	Conventional	HIII headform w/o the neck	45	Side, Front	6
Stigson et al [4]	Carrera Foldable	Conventional	HIII headform w/o the neck	45	Side, Front	6
Stigson et al [4]	Casco Active-TC	Conventional	HIII headform w/o the neck	45	Side, Front	6
Stigson et al [4]	Giro Savant MIPS	MIPS	HIII headform w/o the neck	45	Side, Front	6
Stigson et al [4]	Giro Sutton MIPS	MIPS	HIII headform w/o the neck	45	Side, Front	6
Stigson et al [4]	Hövding 2.0	Hövding	HIII headform w/o the neck	45	Side, Front	6
Stigson et al [4]	Limar Ultralight	Conventional	HIII headform w/o the neck	45	Side, Front	6
Stigson et al [4]	Melon Urban Active	Conventional	HIII headform w/o the neck	45	Side, Front	6
Stigson et al [4]	Occano U MIPS	MIPS	HIII headform w/o the neck	45	Side, Front	6
Stigson et al [4]	Occano Urban	Conventional	HIII headform w/o the neck	45	Side, Front	6
Stigson et al [4]	POC Octal	Conventional	HIII headform w/o the neck	45	Side, Front	6
Stigson et al [4]	POC Octal AVIP MIPS	MIPS	HIII headform w/o the neck	45	Side, Front	6
Stigson et al [4]	Scott Stego MIPS	MIPS	HIII headform w/o the neck	45	Side, Front	6
Stigson et al [4]	Smith Forefront	Koroyd	HIII headform w/o the neck	45	Side, Front	6
Stigson et al [4]	Spectra Urbana	MIPS	HIII headform w/o the neck	45	Side, Front	6
Stigson et al [4]	YAKKAY with cover	Conventional	HIII headform w/o the neck	45	Side, Front	6
Stigson et al [4]	YAKKAY without cover	Conventional	HIII headform w/o the neck	45	Side, Front	6

Table S1: Overview of the literature with relevant kinematic information of the bicycle helmet drop test experiments.

Study	Helmet Model	Mitigation Type	Headform Model	Anvil Angle (°)	Impact Location	Impact Velocity (m/s)
Kurt et al [5]	Hövding 1.0	Hövding	NOCSAE headform on the rigid neck	0	Side	6
Kurt et al [5]	Bell Solar	Conventional	NOCSAE headform on the rigid neck	0	Side	6
Bland et al [6–8]	Bell Solar Flare	Conventional	Magnesium ISO headform on the ball arm neck & NOCSAE headform on the HIII neck	0 & 30	Side, Front	3.4, 6.2 & 5.1, 6.6
Bland et al [6–8]	Bell Star Pro	Conventional	Magnesium ISO headform on the ball arm neck & NOCSAE headform on the HIII neck	0 & 30	Side, Front	3.4, 6.2 & 5.1, 6.6
Bland et al [6–8]	Bell Super 2 MIPS	MIPS	Magnesium ISO headform on the ball arm neck & NOCSAE headform on the HIII neck	0 & 30	Side, Front	3.4, 6.2 & 5.1, 6.6
Bland et al [6–8]	Catlike Whisper	Conventional	Magnesium ISO headform on the ball arm neck & NOCSAE headform on the HIII neck	0 & 30	Side, Front	3.4, 6.2 & 5.1, 6.6
Bland et al [6–8]	Giro Sutton MIPS	MIPS	Magnesium ISO headform on the ball arm neck & NOCSAE headform on the HIII neck	0 & 30	Side, Front	3.4, 6.2 & 5.1, 6.6
Bland et al [6–8]	Giro Synthe	Conventional	Magnesium ISO headform on the ball arm neck & NOCSAE headform on the HIII neck	0 & 30	Side, Front	3.4, 6.2 & 5.1, 6.6
Bland et al [6–8]	Nutcase Watermelon	Conventional	Magnesium ISO headform on the ball arm neck & NOCSAE headform on the HIII neck	0 & 30	Side, Front	3.4, 6.2 & 5.1, 6.6
Bland et al [6–8]	Smith Optics Overtake	Koroyd	Magnesium ISO headform on the ball arm neck & NOCSAE headform on the HIII neck	0 & 30	Side, Front	3.4, 6.2 & 5.1, 6.6
Bland et al [6–8]	Schwinn Trasher	Conventional	Magnesium ISO headform on the ball arm neck & NOCSAE headform on the HIII neck	0 & 30	Side, Front	3.4, 6.2 & 5.1, 6.6
Bland et al [6–8]	S-Works Evade	Conventional	Magnesium ISO headform on the ball arm neck & NOCSAE headform on the HIII neck	0 & 30	Side, Front	3.4, 6.2 & 5.1, 6.6
Bland et al [6–8]	Bell Draft	Conventional	NOCSAE / HIII with & without HIII neck	45	Side, Front	6
Bliven et al [9]	Scott ARX with WaveCel structure	WaveCel	HIII headform on the HIII neck	30, 45, 60	Front	4.8, 6.2
Bliven et al [9]	Scott ARX Plus	MIPS	HIII headform on the HIII neck	30, 45, 60	Front	4.8, 6.2
Bliven et al [9]	Scott ARX	Conventional	HIII headform on the HIII neck	30, 45, 60	Front	4.8, 6.2
Petersen et al [10]	2017 Vivo, Scott without MIPS	Conventional	NOCSAE headform on the HIII neck	45	Side, Front	6.5
Bottlang et al [11]	Scott ARX	Conventional	HIII headform on the HIII neck	45	Front	6.2
Bottlang et al [11]	Scott ARX Plus	MIPS	HIII headform on the HIII neck	45	Front	6.2
Bottlang et al [11]	POC Auric SPIN	SPIN	HIII headform on the HIII neck	45	Front	6.2
Bottlang et al [11]	6dhelmets ATB-1T EVO	ODS	HIII headform on the HIII neck	45	Front	6.2
Bottlang et al [11]	Kali Protectives Tava	Conventional	HIII headform on the HIII neck	45	Front	6.2
Abayazid et al [12]	Bontrager specter WaveCel	WaveCel	HIII headform w/o the neck	45	Side, Front	6.3
Abayazid et al [12]	POC axion SPIN	SPIN	HIII headform w/o the neck	45	Side, Front	6.3
Abayazid et al [12]	POC tectal SPIN	SPIN	HIII headform w/o the neck	45	Side, Front	6.3
Abayazid et al [12]	Hovding 3.0	Hövding	HIII headform w/o the neck	45	Side, Front	6.3

Table S2: Overview of the literature with number of tests and total number of impact or oblique tests for each headform for each mitigation system. *The Ogle headform in [1] was connected to a partial neck which was considered in the no-neck group in our analysis.

Study	Mitigation Type	Model Head Used	Number of tests	Total number of oblique impacts	Total number of radial impacts
[2]	AIM	Magnesium ISO headform on the HIII neck [2]	2	1	1
[1, 3–11]	Conventional	NOCSAE headform on the HIII neck [5–7, 10]	88	34	1
		HIII headform on the HIII neck [3, 4, 6, 9, 11]		30	3
		Ogle headform w/o neck* [1]		4	0
		Magnesium ISO headform on the HIII neck [8]		1	15
[4, 5, 12]	Hövding	HIII headform w/o the neck [4, 12]	5	4	0
		NOCSAE headform on the rigid neck [5]		0	1
[4, 7, 8]	Koroyd	HIII headform w/o the neck [4]	8	2	0
		Magnesium ISO headform on the HIII neck [8]		0	2
		NOCSAE headform on the HIII neck [7]		4	0
[4, 7–9, 11]	MIPS	HIII headform w/o the neck [4, 9, 11]	31	19	0
		Magnesium ISO headform on the HIII neck [8]		0	4
		NOCSAE headform on the HIII neck [7]		8	0
[11]	ODS	HIII headform on the HIII neck [11]	1	1	0
[11, 12]	SPIN	HIII headform on the HIII neck [11, 12]	7	7	0
[9, 12]	WaveCel	HIII headform on the HIII neck [9, 12]	6	6	0

1. Angular momentum

Fig. S1 shows a schematic drawing of the head on an anvil. θ is the angle between the headform and the horizontal axis and α is the anvil angle with respect to the flat floor. In the next step, we calculated the angular momentum for each case. To simplify the calculations, we assumed that the headform is a sphere, the center of gravity of the headform is at a geometrical center of it and the rigid neck has a negligible weight. For group 2 and 3, we calculated the moment of inertia of the headform relative to its center of mass and then translated it to the center of rotation. To calculate the angular momentum, we assumed the headform as a sphere and a neck as a cylinder. Also, we assumed the center of gravity of the head at the geometrical center of the sphere. To simplify the calculations, we neglect the weight of the rigid neck. The angular momentums for no neck, rigid neck and yes neck groups are calculated using equations 1, 2, and 3, respectively:

$$H_{Impact} = (\frac{2}{5}m_H {r_1}^2 + m_H {r_1}^2)\frac{V_{N2}}{r_0} \tag{1}$$

$$H_{Impact} = (\frac{2}{5}m_H{r_1}^2 + m_H{r_2}^2)\frac{V_{N2}}{r_0} \tag{2}$$

$$H_{Impact} = (\frac{2}{5}m_H{r_1}^2 + \frac{m_N r_n^2}{4} + \frac{m_N L_n^2}{12} + M{r_3}^2)\frac{V_{N2}}{r_3} \tag{3}$$

in which m_H is the mass of the head, m_N is the mass of the neck, M is the whole mass of the neck and head system, and r_0, r_1, r_2, r_3, r_n, L_n and V_{N2} are the parameters that are shown in (Fig. S1). In this figure, the headform is positioned at the angle of θ with respect to the horizontal line. V is the impact velocity and V_{N2} is the component of the impact velocity, perpendicular to the head and neck axis.

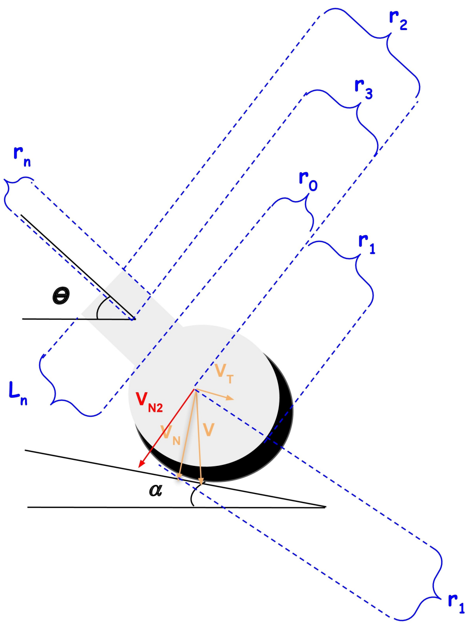

Figure S1: Schematic drawing of the headform on an anvil at the impact point.

References

[1] N. Mills, A. Gilchrist, Oblique impact testing of bicycle helmets, International Journal of Impact Engineering 35 (2008) 1075–1086.

[2] K. Hansen, N. Dau, F. Feist, C. Deck, R. Willinger, S. M. Madey, M. Bottlang, Angular impact mitigation system for bicycle helmets to reduce head acceleration and risk of traumatic brain injury, Accident Analysis & Prevention 59 (2013) 109–117.

[3] P. A. Cripton, D. M. Dressler, C. A. Stuart, C. R. Dennison, D. Richards, Bicycle helmets are highly effective at preventing head injury during head impact: head-form accelerations and injury criteria for helmeted and unhelmeted impacts, Accident Analysis & Prevention 70 (2014) 1–7.

[4] H. Stigson, M. Rizzi, A. Ydenius, E. Engström, A. Kullgren, Consumer testing of bicycle helmets, in: International Research Council on the Biomechanics of Injury Conference (IRCOBI Conference), Antwerp, Belgium, Sept, pp. 13–15.

[5] M. Kurt, K. Laksari, C. Kuo, G. A. Grant, D. B. Camarillo, Modeling and optimization of airbag helmets for preventing head injuries in bicycling, Annals of biomedical engineering 45 (2017) 1148–1160.

[6] M. L. Bland, C. McNally, S. Rowson, Headform and neck effects on dynamic response in bicycle helmet oblique impact testing, in: Proceedings of the IRCOBI Conference. Athens, Greece, pp. 413–423.

[7] M. L. Bland, C. McNally, S. Rowson, Differences in impact performance of bicycle helmets during oblique impacts, Journal of biomechanical engineering 140 (2018).

[8] M. L. Bland, D. S. Zuby, B. C. Mueller, S. Rowson, Differences in the protective capabilities of bicycle helmets in real-world and standard-specified impact scenarios, Traffic injury prevention 19 (2018) S158–S163.

[9] E. Bliven, A. Rouhier, S. Tsai, R. Willinger, N. Bourdet, C. Deck, S. M. Madey, M. Bottlang, Evaluation of a novel bicycle helmet concept in oblique impact testing, Accident Analysis & Prevention 124 (2019) 58–65.

[10] P. G. Petersen, L. V. Smith, D. Nevins, The effect of surface roughness on oblique bicycle helmet impact tests, Proceedings of the Institution of Mechanical Engineers, Part P: Journal of Sports Engineering and Technology 234 (2020) 320–327.

[11] M. Bottlang, A. Rouhier, S. Tsai, J. Gregoire, S. M. Madey, Impact performance comparison of advanced bicycle helmets with dedicated rotation-damping systems, Annals of biomedical engineering 48 (2020) 68–78.
[12] F. Abayazid, K. Ding, K. Zimmerman, H. Stigson, M. Ghajari, A new assessment of bicycle helmets: the brain injury mitigation effects of new technologies in oblique impacts, Annals of biomedical engineering (2021) 1–18.
